# Cost-effectiveness of nivolumab plus ipilimumab as first-line therapy in advanced renal-cell carcinoma

**DOI:** 10.1186/s40425-018-0440-9

**Published:** 2018-11-20

**Authors:** Bin Wu, Qiang Zhang, Jie Sun

**Affiliations:** 1 Medical Decision and Economic Group, Department of Pharmacy, South Campus, Ren Ji Hospital, School of Medicine, Shanghai Jiaotong University, Shanghai, China; 2 Department of Clinical Oncology, Putuo Hospital, Shanghai University of Traditional Chinese Medicine, Shanghai, China; 3 Department of Urology, Ren Ji Hospital, School of Medicine, Shanghai Jiaotong University, Shanghai, China

**Keywords:** Renal cell carcinoma, Nivolumab, Ipilimumab, Sunitinib, Cost-effectiveness

## Abstract

**Background:**

Nivolumab plus ipilimumab improves overall survival and is associated with less toxicity compared with sunitinib in the first-line setting of advanced renal-cell carcinoma (RCC). The current study aimed to assess the cost-effectiveness of nivolumab plus ipilimumab for first-line treatment of advanced RCC from the payer perspectives high- and middle-income regions.

**Methods:**

A decision-analytic model was constructed to evaluate the health and economic outcomes of first-line sunitinib and nivolumab plus ipilimumab treatment associated with advanced RCC. The clinical and utility data were obtained from published reports. The cost data were acquired for the payer perspectives of the United States (US), United Kingdom (UK), and China. Sensitivity analyses were performed to test the uncertainties of the results. Quality-adjusted life-years (QALYs) and incremental cost-effectiveness ratios (ICERs) were used.

**Results:**

Nivolumab plus ipilimumab gained 0.70–0.76 QALYs compared with sunitinib. Our analysis determined the following ICERs for nivolumab plus ipilimumab over sunitinib in first-line advanced RCC treatment: US $ 85,506 /QALY; UK $ 126,499/QALY; and China $ 4682/QALY. Sensitivity analyses found the model outputs to be most affected for body weight and for the prices of nivolumab, sunitinib and ipilimumab.

**Conclusions:**

Nivolumab plus ipilimumab as first-line treatment could gain more health benefits for advanced RCC in comparison with standard sunitinib, which is considered to be cost-effective in the US and China but not in the UK.

**Electronic supplementary material:**

The online version of this article (10.1186/s40425-018-0440-9) contains supplementary material, which is available to authorized users.

## Introduction

The Global Burden of Disease 2015 Study presented that kidney cancer accounted for 1.60% of disease burden associated with neoplasms and ranked 14th in deaths [1, 2]. As the most lethal of the prevalent types of kidney cancer, nearly 30% of patients with renal cell carcinoma (RCC) have locally advanced or metastatic disease at diagnosis because they are generally asymptomatic at disease onset [3, 4]. Over the last decade, targeted agents, such as sunitinib and everolimus, which inhibit the vascular endothelial growth factor receptor (VEGFR) or the mammalian target of rapamycin (mTOR) pathways, have become the standard care for treating advanced RCC. However, despite notable improvements in health outcomes by these new agents, advanced RCC is still incurable with a median overall survival (OS) of 2 years. [5–7] Therefore, it is necessary to develop novel agents for controlling advanced disease.

New agents under the class of immune checkpoint inhibitors have the potential to provide improved survival benefits and improve the quality of life for patients with advanced cancer who previously had few treatment options. [8] These benefits can be gained through antibodies inhibiting the cytotoxic T lymphocyte–associated protein 4 (CTLA-4) or the programmed cell death 1 (PD-1) pathway, either alone or in combination [9, 10]. Recently, nivolumab, a PD-1 inhibitor, has shown survival superiority over everolimus in second-line treatment of metastatic RCC and has been recommended by the clinical guideline [11]. In comparison with sunitinib, the recent CheckMate 214 trial showed nivolumab plus ipilimumab was well tolerated and significantly reduced the risk of death by 32% (hazard ratio [HR] for death, 0.63; *P* < 0.001) for intermediate- and poor-risk patients with advanced or metastatic advanced RCC who were previously untreated in comparison with sunitinib [12]. The nivolumab plus ipilimumab (CTLA-4 inhibitor) strategy was granted U.S. Food and Drug Administration (FDA) approval as a first-line treatment for adults with advanced RCC.

Due to the high cost of immune checkpoint inhibitors, it is unclear whether the promising nivolumab plus ipilimumab treatment would be cost-effective for patients with advanced RCC. One recent systematic review showed the transferability and generalizability of conclusions from the cost-effectiveness analysis are limited because the cost inputs are region-specific [13]. The current study investigated the economic outcomes of introducing nivolumab plus ipilimumab as first-line therapy to the present standard care of patients with advanced RCC in the US, UK and China for the extent of transferability and generalizability, which are the representatives of high- and middle-income regions, respectively.

## Materials and methods

### Model structure

**Fig. 1:**
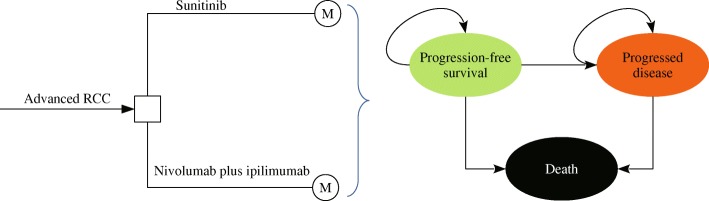
Schematics of the decision tree and the Markov state transition model. RCC: renal-cell carcinoma

A Markov model was developed to evaluate the costs and health outcomes of treating advanced RCC with sunitinib and nivolumab plus ipilimumab. The model included three discrete health states reflecting different characteristics of the disease: PFS, progressed disease (PD) and death (Fig. 1). Because the treatment schedules in CheckMate 214 trial was arranged by using week as the unit, the cycle length of the Markov model was set to be one week [14]. The time horizon was 10-year because 5-year survival rate was lower than 10%, and the initial health state for all of the patients was progression-free survival [15]. During each one-week cycle, the patients either remained in their assigned health state or progressed to a new health state. It was assumed that patients cannot go back to previous health states. The hypothetical patient demographics when entering the model matched those of the patients in the CheckMate 214 trial: 62 years old and 72.8% male, with previously untreated advanced RCC with a clear-cell component [12]. Model development and data analysis were performed in the R statistical environment (version 3.4.2; R Development Core Team, Vienna, Austria).

The main outcomes were expected life years (LYs), quality-adjusted life-years (QALYs) and cost. Cost and QALYs were discounted at an annual rate of 3% in the United States, 3.5% in the United Kingdom and 5% in China [16, 17]. The costs are shown in 2017 US dollars. Incremental cost-effectiveness ratios (ICERs), presented as cost per additional QALY gained, were examined. According to the published literatures, the cost-effectiveness thresholds in US, UK and China were$150,000, $65,000 and $27,351 (3× the per capita gross domestic product of China in 2017), respectively [16, 17].

### Clinical data

Clinical efficacy and safety data were obtained from the CheckMate 214 trial [12]. In intermediate- and poor-risk advanced RCC patients, the 18-month OS rate with nivolumab plus ipilimumab was 75% and was 60% with sunitinib (HR: 0.66, 95% confidence interval [CI]: 0.53–0.82). The median PFS was 11.6 months with nivolumab plus ipilimumab and 8.4 months with sunitinib. According to the results of goodness of fit measured by the *R*^*2*^ statistic, the Weibull survival function *S(t) = exp(−αt*^*β*^*)* and Log-logistic survival function *S(t) = 1/(1 + αt*^*β*^*)* were employed for fitting the Kaplan–Meier PFS and OS probabilities of the sunitinib and nivolumab plus ipilimumab strategy, respectively. The estimated parameters of the Weibull survival function and Log-logistic model are shown in Table 1, and calibration curve showed in Additional file 1. The duration of the PFS and PD phases in two competing strategies was calculated using the area under the PFS and OS survival curves. The difference between the OS and PFS estimated from the parametric survival models was used for calculating the probability from PD to death. [18] After the disease progressed, about 66, 15.8 and 33% of patients in the US, UK and China would receive second-line active treatment according to previous reports [19–21].

**Table 1:** Key clinical and health preference data

Parameters	Values	Reference
Log-logistic survival model of PFS of sunitinib	Scale = 0.01302; Shape = 1.174; r2 = 0.9997	[12]
Log-logistic survival model of PFS of nivolumab plus ipilimumab	Scale = 0.02487; Shape = 0.9312; r2 = 0.9995	[12]
Weibull survival model of OS of sunitinib arm	Scale = 0.00685; Shape = 0.9778; r2 = 0.9939	[12]
Weibull survival model of OS of nivolumab plus ipilimumab	Scale = 0.00414; Shape = 0.9938; r2 = 0.9993	[12]
Probability (%) of total AEs (grade 1 and 2)		
Sunitinib	34 (Range:26–43)	[12]
Nivolumab plus Ipilimumab	47 (Range:59–12)	[12]
Probability (%) of total AEs (grade ≥ 3)		
Sunitinib	63 (Range:47–79)	[12]
Nivolumab plus Ipilimumab	46 (Range:35–58)	[12]
Probability (%) of fatigue (grade ≥ 3)		
Sunitinib	9.2 (Range:6.9–11.4)	[12]
Nivolumab plus Ipilimumab	4.2 (Range:3.2–5.3)	[12]
Probability (%) of hypertension (grade ≥ 3)		
Sunitinib	15.9 (Range:11.9–19.9)	[12]
Nivolumab plus Ipilimumab	0.7 (Range:0.5–0.9)	[12]
Probability (%) of anemia (grade ≥ 3)		
Sunitinib	4.5 (Range:3.4–5.6)	[12]
Nivolumab plus Ipilimumab	0.4 (Range:0.3–0.5)	[12]
Probability (%) of palmar–plantar erythrodysesthesia (grade ≥ 3)		
Sunitinib	9.2 (Range:6.9–11.4)	[12]
Nivolumab plus Ipilimumab	0 (Range:0–0)	[12]
Probability (%) of thrombocytopenia (grade ≥ 3)		
Sunitinib	4.7 (Range:3.5–5.8)	[12]
Nivolumab plus Ipilimumab	0 (Range:0–0)	[12]
Proportion (%) of receiving active second-line treatment		[19–21]
US	66 (Range:7.5–80)	
UK	15.8 (Range:7.5–80)	
China	33 (Range:7.5–80)	
Health preferences		
Utility of PFS	0.78 (Range:0.71–0.849)	[24, 29, 41, 42]
Utility of PD	0.66 (Range:0.45–0.823)	[24, 29, 41, 42]
Disutility due to AEs (grade 1 and 2)	0.014 (Range:0.008–0.02)	[41]
Disutility due to AEs (grade ≥ 3)	0.157 (Range:0.11–0.204)	[41]

*Abbreviations*: *AE*, adverse event; *PD*, progressed disease; *PFS*, progression-free survival; *OS*, overall survival

### Cost and utility estimates

This analysis adopted the third-party payer, the National Health Service and health care perspectives in the US, UK and China, respectively, which only considered direct medical costs, including the first- and second-line treatment, management of treatment-related serious adverse events (SAEs), routine follow-ups and monitoring, best supportive care (BSC) and terminal care (Table 2). For comparability, costs for three countries were reported in 2017 US dollars. GBP and Chinese Yuan were converted into US dollars by using the following exchange formula: 1US $ = GBP 0.7075 and 1US $ = CNY 6.8. US and UK costs associated with health care services were inflated to 2017 values according to the US and UK consumer price index. [22, 23] As previous study done, we therefore adopted the approach of taking the average increase in the index for the previous three years when local index is lack [23]. Because the Chinese health care costs were controlled by the government and kept stable, the Chinese costs were not inflated in the current analysis.

**Table 2:** Cost (US $) estimates (expected value [range])

Parameters	United States	United Kingdom	China
Price of sunitinib per 50 mg	601.9 (301–601.9)^#^ [28]	145.7 (72.87–145.7) ^#^ [35]	275.2 (137.6–275.2) ^#^ [21]
Price of ipilimumab per 50 mg	7324 (3662 – 7324) ^#^ [28]	4875 (2438 – 4875) ^#^ [26]	4655 (2328–7324)^a #^
Price of nivolumab per 100 mg	2670 (1335 – 2670) ^# 31^	1426 (713.1–1426) ^#^ [26]	1362 (680.9–1362) ^#^
Cost of follow-up and monitoring per cycle	422 (348.1–495.8) [30]	75.78 (48.32–103.2) [35]	6.13 (4.9–8.58) [21]
Cost of second-line active treatment per patient	27,936 (26,429 – 29,443) [32]	15,012 (14,793 – 15,231) [35]	21,081 (11,927 – 26,628) [21]
Cost of BSC per cycle	1213 (987–1438) [31]	88.23 (70.53–105.9) [35]	52.53 (49.1–69.21) [39]
Cost of terminal care per patient	10,713 (8570 – 12,856) [24, 32]	10,366 (8566 – 12,849) [19]	1893 (1564–2346) [45]
Cost of managing AEs (grade ≥ 3) per event			
Fatigue	139 (1.06–2018) [32–34]	483.6 (0–967.2) [38]	110.3 (82.72–137.9) [21]
Hypertension	201.9 (1.08–6533) [32–34]	27.3 (0–54.6) [38]	12.35 (9.26–15.44) [21]
Anemia	4638 (3326 – 5949) [32–34]	3242 (3097 – 3388) [38]	508.2 (381.2–635.3) [21]
Palmar–plantar erythrodysesthesia	118.8 (3.43–1748) [32–34]	131.3 (98.48–164.1) [19]	15.21 (8.85–21.57) [40]
Thrombocytopenia	4014 (1716 – 9391) [34]	4927 (4764 – 5091) [38]	3395 (2546 – 4244) [21]
Cost of drug administration per unit	292 (219–365) [16]	405.3 (304–506.7) [26]	17.65 (13.24–22.06) [21]

^a^The prices were assumed by multiplying the price of ipilimumab in UK and the ratio of the price of nivolumab between UK and China

# The ranges were assumed

Based on the CheckMate 214 trial, sunitinib was prescribed at a dose of 50 mg/day for 4 weeks followed by 2 weeks off treatment [12]. Nivolumab and ipilimumab were administered intravenously at a dose of 3 mg/kg and 1 mg/kg, respectively, every 3 weeks for four doses (induction phase), followed by nivolumab monotherapy at a dose of 3 mg/kg every 2 weeks (maintenance phase). To calculate the dosage of nivolumab and ipilimumab agents, we assumed a typical patient weighed 71.4 kg in the US, 78.7 kg in the UK and 59 kg in China, and the range (29–112 kg) was used in the sensitivity analysis [16, 24, 25]. Based on previous reports, the maximum treatment duration of nivolumab plus ipilimumab was two years [16, 26]. The medication cost of sunitinib would be adjusted because the actual dosage intensity of sunitinib was 67% of the planned dosage [27]. The prices of sunitinib, nivolumab and ipilimumab in the US (Average Wholesale Price) and UK were collected from public databases and literature, respectively [26, 28]. The Chinese price of nivolumab is $1362/100 mg. Because ipilimumab are still unavailable in the Chinese market, we estimate its price by multiplying the price of ipilimumab in UK and the ratio of the price of nivolumab between UK and China. Other cost data were collected from published literature [19, 21, 23, 24, 26, 28–35].

The analysis included grade 3/4 adverse events (AEs) that had notably different probabilities between the arms of the CheckMate 214 trial: fatigue, hypertension, anemia, palmar–plantar erythrodysesthesia and thrombocytopenia [12]. The recommended management of AEs might be found in the clinical guidelines [36, 37]. The costs of managing AE per event in the US, UK and China were extracted from literatures. [19, 21, 32–34, 38–40]

Mean health utility scores for PFS and PD state were derived from published literature (Table 1) [24, 29, 41, 42]. The disutility values due to grade 1/2 and 3/4 AE were included in this analysis [41].

### Sensitivity analysis

One-way and probabilistic sensitivity analyses were used to test the uncertainty in the model. In the one-way sensitivity analyses, to identify key model input parameters that had substantial impact on the model outcome, relevant parameters were adjusted one-by-one to their respective low and high values, which are listed and illustrated in Tables 1 and 2. The ranges of the parameters used in the one-way sensitivity analyses were obtained from the published literature; when reported data were not available, a range of ±25% of the base-case value was used. An assumed 50% discount of the price of sunitinib, ipilimumab and nivolumab were used for one-way sensitivity analyses. The results of the one-way sensitivity analyses are presented in a Tornado diagram. For the probabilistic sensitivity analyses (PSA), parameters were sampled using the Monte Carlo method to run 1000 replicated outcomes. Based on the ISPOR-SMDM Modeling Good Research Practices Task Force report on model parameter estimation and uncertainty, the values of the input parameters were sampled from lognormal distributions for costs and from β distributions for utility values and probabilities or proportions with an assumed standard deviation of 25% from mean values [43]. The price of sunitinib, ipilimumab and nivolumab were fixed in the PSA due to branded drugs. Cost-effectiveness acceptability curves were generated to present the probabilities of cost-effectiveness.

## Results

### Base-case analysis

For the advanced RCC patients, nivolumab plus ipilimumab instead of sunitinib provided an additional 1.17 life years. Compared with the sunitinib strategy, the mean incremental costs and QALYs of the nivolumab plus ipilimumab were $ 65,114 and 0.76, $ 94,356 and 0.75 and $3286 and 0.70 for the population in the US, UK and China, respectively. The ICERs for the nivolumab plus ipilimumab versus the sunitinib were $ 85,506 in the US, $126,499 in the UK and $4682 in China (Table 3).

**Table 3:** Summary of Cost ($) and Outcome Results in the base-case analysis

Strategy	Cost	Expected LYs	QALYs	ICER^a^	Comments
United States					
Sunitinib	297,693	3.01	2.04	NA	Cost-effective
Nivolumab plus ipilimumab	362,807	4.18	2.80	85,506	
United Kingdom					
Sunitinib	75,034	3.01	2.02	NA	Not cost-effective
Nivolumab plus ipilimumab	169,390	4.18	2.77	126,499	
China					
Sunitinib	97,846	3.01	1.96	NA	Cost-effective
Nivolumab plus ipilimumab	101,132	4.18	2.66	4682	

^a^Incremental cost per *QALY. LY*, life years; *QALY*, quality-adjusted life-years

### Sensitivity analysis

**Fig. 2:**
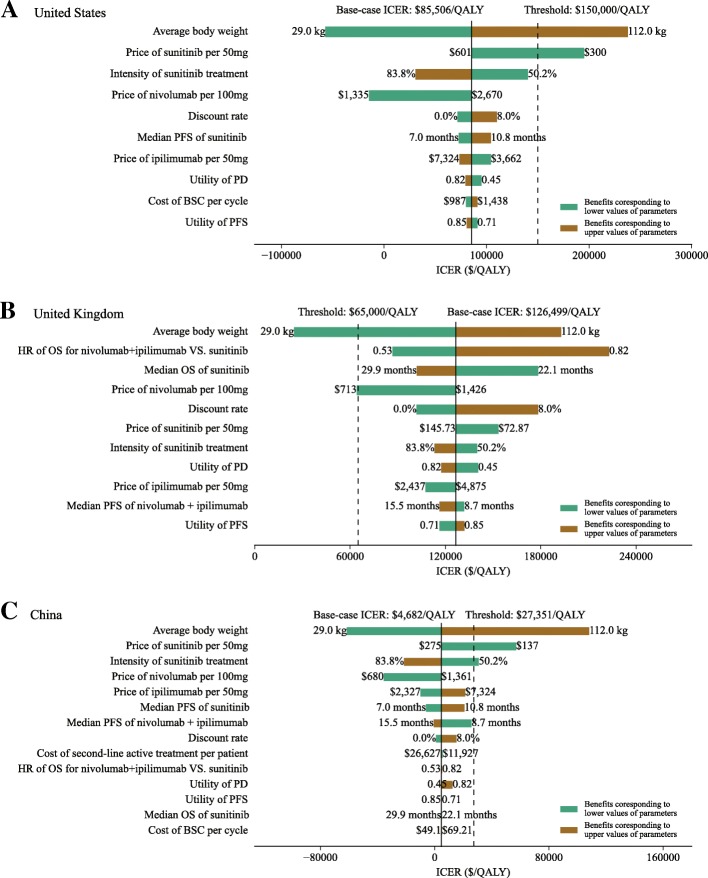
One-way sensitivity analyses of nivolumab plus ipilimumab in comparison with sunitinib in United States (**a**), United Kingdom (**b**) and China (**c**)

The one-way sensitivity analyses revealed that the results of the model were more sensitive to body weight because this variable had the greatest impact on ICER, which showed that the nivolumab plus ipilimumab strategy would become more favorable as the body weight decreased (Fig. 2). Other considerable influential parameters in the US were the prices of sunitinib and nivolumab, in the UK were the HR of OS for nivolumab plus ipilimumab versus sunitinib, the median OS time of sunitinib treatment and the prices of nivolumab, and in China were the prices of nivolumab, sunitinib and ipilimumab. Model results were robust to changes in other model inputs, including the cost of second-line active treatment, costs and disutilities related to AEs.

**Fig. 3:**
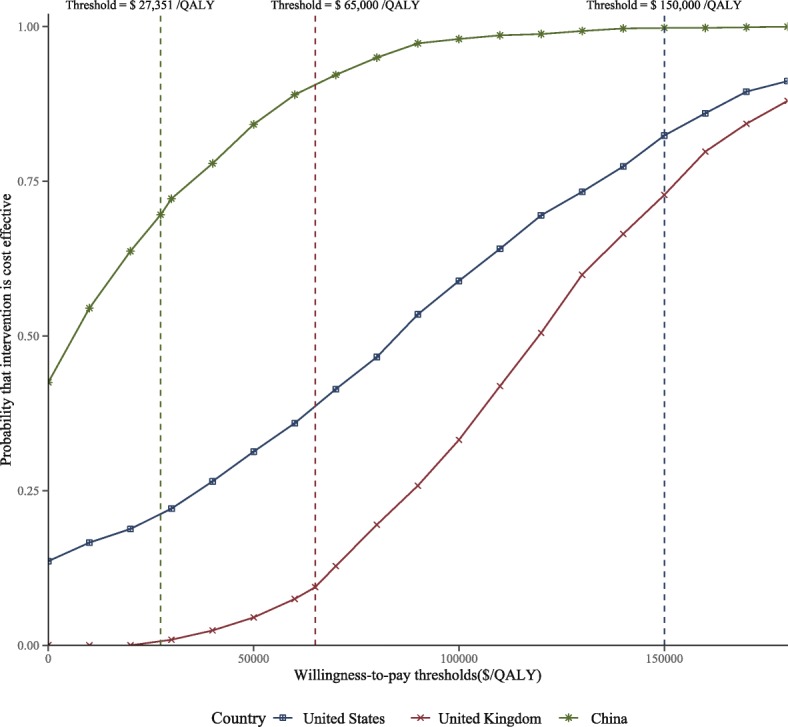
The cost-effectiveness acceptability curves for nivolumab plus ipilimumab strategy compared to the sunitinib strategy in three countries

Compared to the sunitinib strategy, the cost-effectiveness acceptability curves showed that the nivolumab plus ipilimumab produced nearly 80.1, 9.2 and 65.2% probabilities of cost-effectiveness when the threshold was equal to $150,000, $65,000 and $27,351 in the US, UK and China, respectively (Fig. 3).

## Discussion

Reports of a clinical benefit from nivolumab plus ipilimumab therapy in a clinical study caused great excitement among both oncologists and patients [12]. However, the widespread use of these agents comes with a sharp increase in health resource consumption, which is of concern to clinicians and administrators [44]. This evaluation first investigated the cost-effectiveness of nivolumab plus ipilimumab for patients with newly diagnosed advanced RCC, and our results are of great significance in high- and middle-income settings. The results suggested that the nivolumab plus ipilimumab therapy produced an additional 0.66 life years and > 0.70 QALYs compared with standard sunitinib treatment with a substantial augment of cost, leading to average incremental cost-effectiveness ratios of $85,880/QALY in the US, $126,483/QALY in the UK and $9866 /QALY in China. At a willingness-to-pay threshold values of $150,000, $65,000 and $27,351 per QALY gained in the US, UK and China, this main finding indicated that the nivolumab plus ipilimumab strategy was a cost-effective therapeutic approach in the US and China, but not in the UK. The acceptability curve also demonstrated this finding that a majority of certainty was achieved by nivolumab plus ipilimumab at the threshold of $150,000 in the US and $27,351/QALY in China, and a paucity of certainty at the threshold of $65,000/QALY in UK, respectively.

One recent publication reported the results of economic evaluations of nivolumab as second-line therapy for advanced RCC from the payer perspective of the US [16], which presented an ICER of $146,532/QALY versus everolimus that indicated that nivolumab is cost-effective. However, another economic study found that second-line immunotherapy with pembrolizumab versus chemotherapy for bladder cancer is cost-effective in the US, but not in the UK, Canada and Australia. These results are similar to our findings for the US and UK. The potential reason might be the gaps of the prices of sunitinib, nivolumab and ipilimumab between the US and UK. We noted that the price of sunitinib in the US is about four times that in the UK, and nivolumab and ipilimumab are about two times as much. This gap leaded the incremental cost of nivolumab plus ipilimumab against sunitinib to be higher in UK ($94,356) than in US ($ 65,114), which yielded the unfavorable results in UK and favorable results in US. Because the drug prices varied across the different regions due to local affordability and market assess scheme, the economic evaluation needs to consider a diversity of health settings for easy transferability among different regions.

The results of one-way sensitivity analysis found that body weight had the greatest impact on the model outcome. One recent study that evaluated the cost-effectiveness of nivolumab versus everolimus in patients with advanced RCC in the US also found that average body weight had the greatest impact on the ICER for nivolumab versus everolimus (base case US $51,714; range US $8863–$94,566) [24]. The potential reason for this is that the dosage of sunitinib treatment is administered regardless of body weight, and the dosage of nivolumab and ipilimumab need to be adjusted according to the body weight. More dosages of nivolumab and ipilimumab are needed in patients with high body weight, which might increase barriers to affordability. We suggest that nivolumab and ipilimumab might be paid for per patient or per treatment cycle instead of per vial.

Our analyses have several weaknesses. First, modeling with Weibull and Log-logistic function to project long-term PFS and OS beyond the observational time of the trial is an inevitable limitation of this study. Second, this trial-based study could not wholly track the natural disease course in the real world. This approach could not adequately reflect effectiveness and resource consumption in routine clinical practice. Third, the present study did not solely consider nivolumab monotherapy as second line treatment after the disease progressed because it is only one of alternatives in the subsequently systemic therapy due to their comparable efficacy [4], whose cost also only had a paucity of impact in sensitivity analyses. Fourth, we did not measure the budget impact of nivolumab plus ipilimumab treatment on society. Wide prescription of these agents might intensively raise the financial burden. Fifth, the costs of grade 1/2 AEs were excluded and the impact of immune-related AEs (irAEs) were did not solely considered due to no evidence indicating the notably difference of managing irAEs and non-irAEs, which might underestimate or overestimate the benefits of nivolumab plus ipilimumab. Fortunately, economic outcomes were not sensitive to all parameters related to AEs. Sixth, due to the absence of head-to-head trials, the current analysis did not include other competing agents, such as pazopanib that is a cost-effective option in comparison with sunitinib [27, 35, 41]. The current study needs to be updated by including these novel competing alternatives. Finally, due to the heterogenicity of payer perspectives, such as the mixed public and private payer in the US, the finding should be conservatively generalized to other regions. However, because the findings of this evaluation reflected the general clinical conditions of managing advanced RCC, it might be a valuable reference for decision-makers.

In conclusion, in patients with advanced RCC in the contexts of the US and China, nivolumab plus ipilimumab is likely to be cost-effective in these countries, but not in the UK.

## Additional file

Additional file 1: Calibration curve: progression-free and overall survival. Predicted data (dotted line) were plotted along with the observed data from CheckMate 214 trial (solid line). (PDF 38 kb)

## Abbreviations

AEs: Adverse events
BSC: Best supportive care
CTLA-4: cytotoxic T lymphocyte–associated protein 4
FDA: Food and Drug Administration
HR: Hazard ratio
ICERs: Incremental cost-effectiveness ratios
mTOR: Mammalian target of rapamycin
PD: Progressed disease
PD-1: Programmed cell death 1
PSA: Probabilistic sensitivity analyses
QALYs: Quality-adjusted life-years
RCC: Renal-cell carcinoma
SAEs: Serious adverse events
UK: United Kingdom
US: United States
VEGFR: Vascular endothelial growth factor receptor
