# Supplementary material for: Cost-effectiveness of nivolumab plus ipilimumab as first-line therapy in advanced renal-cell carcinoma
Source: J Immunother Cancer. 2018 Nov 20;6:124. doi: 10.1186/s40425-018-0440-9 (PMC6247499; doi:10.1186/s40425-018-0440-9)
Supplement: Supplementary file 1 — Calibration curve: progression-free and overall survival. Predicted data (dotted line) were plotted along with the observed data from CheckMate 214 trial (solid line). (PDF 38 kb) [file 40425_2018_440_MOESM1_ESM.pdf]

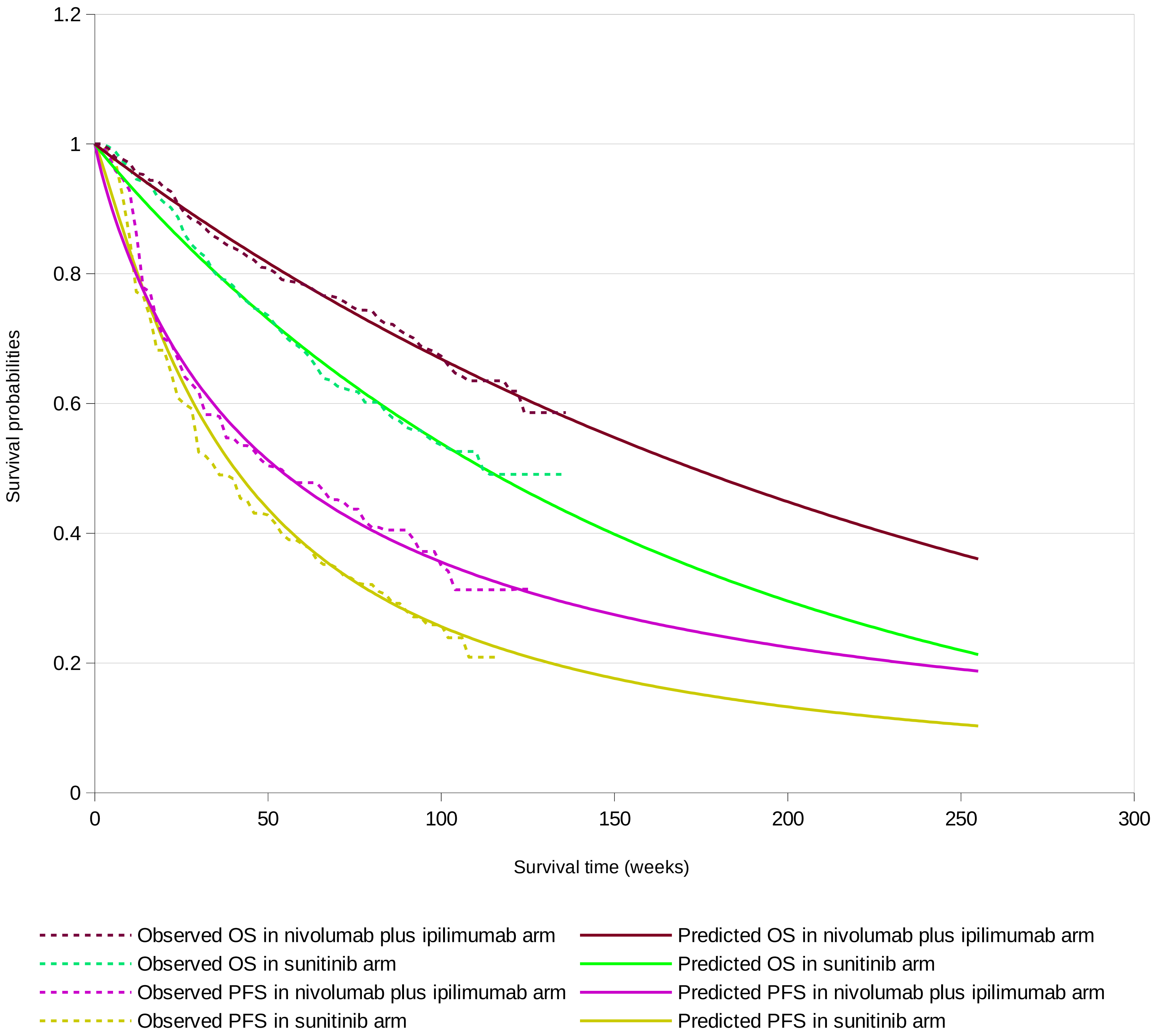

Additional file 1. Calibration curve: progression-free and overall survival. Predicted data (dotted line) were plotted along with the observed data from CheckMate 214 trial (solid line).
